# Tuberculosis diagnostic networks: Moving beyond the laboratory to end tuberculosis in Africa

**DOI:** 10.4102/ajlm.v6i2.608

**Published:** 2017-03-29

**Authors:** Amy Piatek

**Affiliations:** 1 Bureau for Global Health, United States Agency for International Development, Washington, DC, United States

Tuberculosis is a curable disease when diagnosed early and treated with an appropriate drug regimen, yet 750 000 Africans die unnecessarily every year from tuberculosis. We know that when a person with tuberculosis is treated in Africa, 81% will be cured – and even those infected with HIV are cured most of the time. Tragically, less than half of all Africans with tuberculosis are diagnosed and started on treatment. This vastly low tuberculosis case detection contributes to unacceptably high rates of death from tuberculosis in Africa. Deaths attributed to tuberculosis, among both HIV-negative and HIV-positive people in Africa, are higher than any region in the world – more than twice the global rate among HIV-negative people, and five times higher among people with HIV. The need for more accurate, faster technologies to diagnose tuberculosis in sub-Saharan Africa is more urgent than ever before. In 2015, 2.7 million Africans fell ill with tuberculosis (30% of whom were infected with HIV); however, only 48% sought and received a diagnosis and were reported to national tuberculosis programmes. Consequently, over 1.4 million people were undiagnosed and likely transmitted tuberculosis to their families and communities for weeks and months, until their probable death. Of these undiagnosed cases, more than 80 000 have multi-drug resistant tuberculosis. Although undiagnosed tuberculosis is a small proportion of the overall missing cases, increasing prevalence of multi-drug resistant tuberculosis will eventually diminish Africa’s current successes toward stopping the tuberculosis epidemic.^1^

Even if all Africans with presumptive tuberculosis or multidrug-resistant tuberculosis accessed a diagnostic facility, the predominant testing method using smear microscopy will accurately detect tuberculosis in 20% to 80% of tuberculosis cases, depending on the report and method used.^2^ Since smear microscopy can only detect tuberculosis in specimens with sufficient bacillary load and does not identify resistance to anti-tuberculosis drugs, the method is of very limited utility for diagnosing HIV-associated tuberculosis^3^ and is of no use in diagnosing a person with drug-resistant tuberculosis. Other methods to diagnose active tuberculosis and drug-resistant tuberculosis are available, including culture-based tools for diagnosis and drug susceptibility testing, nucleic acid amplification technologies and sequencing methods, lateral flow-based rapid diagnostic tests, and detection of volatile organic compounds.

The success of a tuberculosis testing method not only relies on the test’s diagnostic accuracy but on whether or not it is placed in the context of a comprehensive and functional tuberculosis diagnostic network. Even if African national tuberculosis programmes have access to the full menu of new and old testing methods, significant improvements in tuberculosis case detection will not happen if underlying systems that make up the diagnostic network are absent. A comprehensive and high-quality tuberculosis diagnostic network is one where tests are accessible, accurate, adaptable, and rapid:

- *Accessibility:* The network is based on a patient-centred approach that consists of the appropriate diagnostic tests (based on disease epidemiology) that are available where people seek care (e.g., all levels of the system in the public or private sector).
- *Accuracy:* The network provides quality-assured results that give clinicians, laboratorians and the community confidence to diagnose tuberculosis and multi-drug resistant tuberculosis and place a person on appropriate treatment.
- *Adaptability:* The network provides a strong platform to support all diagnostic tests currently used by a national tuberculosis programme and tests that will be introduced in the future.
- *Rapidity:* The network consists of tests that produce results quickly (hours instead of days) – or supports a network to reduce the turn-around time from specimen collection to reporting of results to initiation of treatment for longer duration tests.

To reach an optimal diagnostic network, immediate investments will be needed in several supportive areas such as specimen transport and referral mechanisms; electronic linkages between diagnostic and clinical facilities; laboratory, programme and clinical staff capacity building; commodity and logistics management; biosafety policies and procedures; and continuous quality improvement schemes. The African region will need to continue to develop and implement supportive policies and initiatives for the development of tuberculosis diagnostic networks and systems strengthening. One such initiative is the Global Laboratory Initiative for Africa (GLI Africa), established in 2014 to support countries to achieve quality assured, accessible and sustainable tuberculosis laboratory services in the African Region. GLI Africa assists countries through its network of national tuberculosis programmes and laboratories, multilateral agencies, development and technical partners, public health institutions and other key stakeholders to improve tuberculosis laboratory and diagnostic services through several areas of support including:

- Restructuring the *laboratory* network to a patient-centred, coordinated *diagnostic* network;
- Narrowing the gap between policy and implementation;
- Framing continuous quality improvement within the diagnostic network;
- Establishing the diagnostic-clinical interface;
- Implementing biosafety and biosecurity in the network and building bioengineering capacity; and
- Building capacity and mechanisms for responsive technical assistance.

This issue of the *African Journal of Laboratory Medicine* explores how countries are optimising current diagnostics, introducing and scaling up modern technologies, and thinking through how to improve the many health system factors that are preventing the successful uptake and use of many of the available diagnostics. While Africa and the entire global community continues to need developers interested in creating the next best diagnostic, countries must adapt and improve current systems to maximise the use of existing technologies. No African should die of tuberculosis because they cannot access a fast and reliable test that leads them to life-saving treatment.
